# Rethinking Negative Reinforcement in Zoo Animal Welfare: A Constructional Approach to Distance as a Reinforcer to Address Fear and Aggression

**DOI:** 10.3390/ani16142206

**Published:** 2026-07-16

**Authors:** Barbara Heidenreich, Annette Pedersen

**Affiliations:** 1 Animal Training Fundamentals, Austin, TX 78745, USA; 2 Copenhagen Zoo, Roskildevej 38, 2000 Frederiksberg, Denmark; ap@zoo.dk

**Keywords:** negative reinforcement, constructional approach, animal welfare, fear responses, aggressive behavior, genuine choice, zoo animal training, systematic desensitization, counterconditioning, functional reinforcement

## Abstract

Fear and aggressive responses in zoo animals are commonly maintained by negative reinforcement, with the animal’s behavior functioning to create distance from an aversive stimulus. Traditional approaches such as systematic desensitization and counterconditioning may be less effective because they do not contact this maintaining contingency and may instead superimpose competing contingencies, producing incomplete change. This paper presents the case for negative reinforcement shaping as a functionally aligned, welfare-positive intervention applied within the constructional approach. We review the empirical literature, address persistent misconceptions, and provide procedural guidance. Two case studies are presented: a male Amur tiger (*Panthera tigris altaica*) at Copenhagen Zoo whose aggressive responses toward a specific caregiver changed to calm, affiliative responding across 42 min of training, enabling subsequent positive reinforcement-based medical care; and an orangutan (*Pongo pygmaeus*) at Dublin Zoo whose avoidance of a suddenly aversive night holding area was resolved within approximately 15 min, resulting in calm, sustained presence with the door closed. Genuine choice, contingency analysis, and the transition from negative to positive reinforcement are discussed. We conclude that negative reinforcement shaping within the constructional approach may provide a useful, function-based alternative for distancing-maintained behavior and is accessible to practitioners with foundational training.

## Simple Summary

Fear and aggressive behaviors are among the most challenging presentations in zoo animal management and carry significant welfare implications for the animals experiencing them. These behaviors are frequently maintained by negative reinforcement: the animal’s behavior creates distance from an aversive stimulus or conditions. Traditional approaches such as systematic desensitization and counterconditioning may produce limited outcomes because they do not directly address the escape or avoidance contingency maintaining the behavior. This paper introduces the systematic application of negative reinforcement procedures as a functionally aligned, potentially welfare-positive alternative for behaviors maintained by distancing contingencies. These welfare-positive qualities are not inherent to negative reinforcement itself; they emerge from its application within the constructional approach, supported by Nonlinear Contingency Analysis and genuine choice through increased degrees of freedom. Drawing on this framework, we present two case studies: a male tiger’s aggressive responses toward a caregiver at Copenhagen Zoo, and an orangutan’s avoidance of a night holding area that had unexpectedly become associated with an aversive event at Dublin Zoo. We argue that understanding and applying negative reinforcement within the constructional approach, when behavior is maintained by distancing contingencies, is within reach of the regular practitioner and may meaningfully contribute to zoo animal welfare practice.

## 1. Introduction

Fear and aggressive responses are among the most consequential behavioral challenges in zoo animal care. When animals consistently avoid caregivers, lunge at enclosure barriers, emit alarm vocalizations at routine procedures, or refuse to shift between spaces, these behaviors can constrain veterinary care, compromise staff safety, and, most importantly, may indicate that the animal is experiencing aversive conditions. For the animal, these behaviors represent competent attempts to control an aversive environment. They are not pathologies. They are functional responses to functional contingencies [1,2,3].

A key insight from behavioral science is that many of these presentations share a common maintaining contingency: negative reinforcement. The animal’s behavior—moving away, charging, growling, refusing to shift—may be maintained by negative reinforcement: it reliably produces distance from, or removal of, an aversive stimulus. Once caregivers recognize this, a correspondingly functional approach to intervention becomes available: use the removal of that aversive stimulus—distance—as the reinforcer to shape new, more desirable responses [4].

Despite this, the dominant intervention model in zoo animal training continues to rely on systematic desensitization and counterconditioning. These approaches, while familiar and widely taught, operate through fundamentally different mechanisms: they attempt to extinguish or override fear responses by pairing aversive stimuli with appetitive ones—a process that, when applied without addressing the underlying contingency, functions as superimposition rather than true counterconditioning. This paper argues that these approaches frequently fail to contact the maintaining contingency precisely when the behavior is maintained by distance or escape. They layer a new contingency on top of an existing one—a process called superimposition—leaving the underlying distancing contingency intact [5,6].

In contrast, procedures that directly target the functional reinforcer—distance—may produce rapid, welfare-positive, and durable outcomes when implemented appropriately and when distance is the relevant functional reinforcer. This approach, grounded in the constructional framework developed by Goldiamond [1] and extended by Layng, Andronis, and Rosales-Ruiz [2,7,8,9,10], has been applied with documented success across species and contexts. Yet it remains underutilized in zoo settings, and several factors may contribute to this: the word “negative” carries unfortunate connotations in everyday language; practitioners trained in positive reinforcement-dominant paradigms may not have encountered this approach in a rigorous form; and the procedural nuances—knowing when to provide distance, how far, for how long, how to raise criteria, and how to read subtle behavioral signals—require specific skill development.

This paper aims to address each of these barriers. We provide a conceptual foundation for the use of negative reinforcement procedures with distancing-maintained behavior, situate this approach within the broader constructional approach framework, review the empirical evidence base, directly address common myths and misconceptions, and offer detailed procedural guidance illustrated by two substantive case studies from zoological settings. We also discuss the important links between this approach, genuine choice, and emotional welfare. Our goal is to make this approach more accessible, more accurately understood, and more widely applied in the service of the animals in our care.

## 2. Background: Fear, Aggression, and the Distancing Contingency

### 2.1. Understanding Behavior Function: What Are These Animals Telling Us?

When an animal moves away from a caregiver, charges the fence, vocalizes in apparent alarm, or refuses to shift into a particular space, we receive important behavioral information. Behavior does not occur in a vacuum; it is shaped and maintained by its consequences. These behaviors may be maintained by negative reinforcement when they reliably produce distance or removal of exposure to aversive conditions—a function that should be determined through observation and assessment rather than assumed from the appearance of the behavior alone [4].

The constructional approach, introduced by Goldiamond [1] and further developed by Layng and Andronis [2,7], provides a framework for understanding these behaviors not as deficits or pathologies to be eliminated, but as competent responses to existing contingencies. While the foundational work was conducted primarily with human populations and in laboratory settings, its extension to animal training contexts has grown substantially over the past two decades [4,9,10,11,12,13]. As Goldiamond observed, what appears to be problematic behavior is often “a competent operant, maintained by the environmental reinforcers it produces, but presently producing these at high cost.” [1] (p. 158). For zoo animals, aggression, avoidance, and resistance may be the most effective behavioral strategies available to the animal under certain environmental contingencies.

The constructional approach invites practitioners to ask not “how do we stop this behavior?” but rather “what does this behavior tell us about what this animal needs, and how can we provide a less costly pathway to the same outcome?” [1,2,14]. This reframe is fundamental. It transforms intervention from suppression of undesired responses to construction of desired functional alternatives.

Goldiamond [1] articulates a framework for understanding emotions as contingency descriptors rather than internal states: emotions track the contingency requirements the animal faces. Layng [15] further develops this framework: fear describes a contingency in which moving away from an aversive stimulus is maintained by negative reinforcement, while aggression describes a contingency in which driving the aversive stimulus away is maintained by negative reinforcement. In this framework, it is the emotions—not the resulting emotional behaviors—that function as descriptors of these contingencies; emotional behavior may itself become an operant maintained by its own consequences, distinct from the contingency it originally reflected. Understanding emotions as contingency descriptors rather than causes of behavior shifts the focus appropriately onto modifying the contingencies themselves rather than attempting to change an internal emotional state directly.

### 2.2. Use of Negative Reinforcement in Zoo Animal Training: Historical Context

The use of negative reinforcement as a deliberate, welfare-oriented procedure in animal training has a relatively recent but substantive history. The foundational scientific work was conducted across several decades, beginning in the 1950s, by Goldiamond, Layng, Andronis, and colleagues [1,2,7] primarily with human populations and pigeons, establishing the theoretical basis for contingency-based approaches that would later be extended to animal training contexts.

The most significant inflection point for animal training applications came through the work of Rosales-Ruiz and his students at the University of North Texas, who pioneered what became known as Constructional Aggression Treatment (CAT) and related procedures, applying negative reinforcement shaping to dogs and other species displaying behaviors maintained by distancing contingencies [10,11]. This body of work produced no fewer than 14 empirical studies exploring the application of constructional approaches with animals: graduate theses from Rosales-Ruiz’s lab at the University of North Texas by Snider, Rentfro, McGee, Ward, and Hardaway [10,12,16,17,18]; a graduate thesis by Plass [19] at Virginia Polytechnic Institute and State University; and peer-reviewed publications by Fernandez and Katz and Rosales-Ruiz [9,13]. These studies documented the effectiveness of using distance as a reinforcer to modify avoidance and aggressive responses across species, including dogs, cats, sheep, iguanas, and horses.

This body of evidence laid the groundwork for expanding these applications into zoological settings, where the range of species, enclosure constraints, and care requirements introduce both unique challenges and compelling opportunities. Heidenreich and Pedersen [4] have been among the primary contributors to translating these principles into zoo practice, with documented case-study applications across taxa published in peer-reviewed and professional forums. Practitioner interest in applying these approaches in zoo settings appears to be increasing, though additional controlled research is needed. This need is beginning to be addressed: a recent multi-subject case series with three tigers (*Panthera tigris*) provides welcome empirical support for distance as a functional negative reinforcer in large felids, documenting the systematic use of trainer withdrawal to resolve generalized aggressive responses and successfully transition animals toward engagement with positive reinforcement [20]. This work represents a valuable and timely contribution to the zoological literature that has, until now, relied heavily on individual case reports.

It is important to recognize that, in our observation, some practitioners in the zoo field appear to have been using some form of negative reinforcement shaping for years, often without labeling it as such, or while mislabeling it as systematic desensitization or counterconditioning. This is not a criticism but an observation with important implications: the procedure has intuitive appeal and functional logic that practitioners often discover through their own experience. The goal of this paper is to make that implicit knowledge explicit, refined, and principled, so that it can be applied with greater consistency, greater effectiveness, and greater welfare benefit.

## 3. Limitations of Traditional Approaches When Distancing Contingencies Are Present

### 3.1. Systematic Desensitization: Extinction-Based and Non-Contingent

Systematic desensitization and counterconditioning are established treatments with documented effectiveness across a range of applications, including exposure-based interventions for medical-procedure-related fears [21]. In applied settings, however, where escape is not experimentally prevented, any window for their unmodified use is narrow: once an escape response occurs and succeeds, it is reinforced, and the behavior becomes operantly maintained by a distancing contingency—the condition addressed throughout the remainder of this paper.

Systematic desensitization was developed by Wolpe in the 1950s as an exposure-based treatment for anxiety disorders [22]. In its classical form, it involves three components: progressive relaxation training, development of a fear hierarchy, and graduated exposure to feared stimuli while the learner maintains a relaxed state. The intended mechanism is extinction: the anxiety or fear response diminishes through repeated non-reinforced exposure [21,22]. Counterconditioning, typically used alongside desensitization, involves pairing the aversive stimulus with an appetitive stimulus to change the stimulus’s function from aversive to neutral or appetitive [23].

A recent systematic review by Abdel-Jalil, Baldwin, and Leaf [21] examined 62 published articles spanning 1968–2021 in which exposure treatments were applied to fears and phobias related to medical procedures, involving 715 participants. This review is particularly instructive for zoo practitioners because the behavioral challenges it addresses—strong fear and avoidance responses that prevent participation in necessary medical care—parallel those often encountered in zoo animal management. While this review was conducted with human participants, the functional principles it identifies—specifically the role of escape and avoidance contingencies in maintaining fear-related behavior—are not species-specific. The behavioral mechanisms of negative reinforcement operate across taxa, and the parallels between human medical fear and zoo animal resistance to veterinary procedures are both conceptually and practically similar. This is not merely a theoretical claim of cross-species relevance: an example of an animal analogue study directly testing Wolpe’s systematic desensitization procedure found that rats receiving graduated exposure to an aversive stimulus paired with eating were significantly less effective at eliminating avoidance than rats given graduated exposure alone, and no more effective than untreated controls, suggesting that the presence of an appetitive stimulus can, at least under some conditions, reduce an animal’s functional exposure to, and processing of, the aversive stimulus it is meant to be paired with [24]. While this is a single study with its own scope and limitations, it offers a non-human empirical data point consistent with the procedural concerns raised by the human-focused review above, lending some additional weight to the argument that these vulnerabilities are not confined to a single species or population. The review found that while exposure treatments produced terminal behavioral outcomes (completion of the necessary medical procedure) in the majority of case study articles, outcomes in group and single-subject designs were more variable, with 10 of 17 group-design studies reporting only partial success. Crucially, the review also identified significant methodological inconsistencies across studies: more than half did not provide the fear hierarchies used, nearly two-thirds did not define behavioral criteria for progression within the hierarchy, and social validity data were absent in nearly half of the studies [21]. These gaps make replication difficult and may mask the degree to which practitioners were unknowingly incorporating operant contingencies into ostensibly exposure-based procedures.

This last point is particularly significant. Abdel-Jalil et al. [21] note that the procedures in many reviewed articles suggest operant involvement despite being framed as exposure treatments: the blocking of safety behaviors, the use of escape extinction, and other features point to negative reinforcement as a maintaining contingency for the phobic behavior—yet few studies explicitly employed procedures designed around operant relations. The authors conclude that a new form of exposure therapy that explicitly addresses the operant contingencies maintaining behavior may produce better outcomes, and they identify negative reinforcement-based constructional procedures as a promising direction for future research [21]. Layng and Abdel-Jalil [25] have termed this approach Constructional Exposure Therapy (CET)—a label that captures both its constructional foundation and its distinction from conventional exposure-based methods. Miller and Abdel-Jalil [26] provide a detailed account of CET’s theoretical foundations and recent case applications, including the resolution of severe food aversion and vacuum fear in individuals with autism, further demonstrating the approach’s effectiveness and generality across populations and behavioral presentations. The procedures described in this paper represent a proposed extension of CET principles to zoo animal training contexts. CET has not yet been formally validated in non-human zoo animals through prospective controlled studies; this paper contributes to that translational effort by documenting two applied cases and situating them within the CET framework.

Several additional features of traditional systematic desensitization create particular challenges in zoo animal practice when the behavior is maintained by a distancing contingency. True systematic desensitization is non-contingent: the procedure does not change in response to the animal’s behavior. The stimulus is presented at a fixed distance, and the practitioner waits for the fear response to extinguish, regardless of the animal’s behavioral signals. In practice, if a practitioner’s behavior changes contingent on the animal’s behavior—moving closer or farther depending on what is observed—they are not doing systematic desensitization; they are shaping. This distinction matters because it changes which procedure is in use and, therefore, what its likely effects and limitations are.

Extinction-based procedures also carry well-documented risks, including frustration and extinction-induced aggression, the possibility of sensitization rather than habituation under poorly controlled conditions, and the risk of spontaneous recovery of the fear response after an apparent success [2,7]. Even when extinction appears successful, the underlying contingency that maintained the fear response has not been addressed; the animal has simply ceased emitting a response that was no longer effective under the specific training conditions. When conditions change, the original behavior is likely to return. This is consistent with what practitioners report: the process is often slow, requiring many sessions with frequent regression [21,25].

### 3.2. Counterconditioning and the Problem of Superimposition

Counterconditioning, when applied rigorously, involves simultaneous contact with the aversive and appetitive stimuli with the intent of changing the stimulus function of the aversive. Even where the behavior is classically conditioned, however, research suggests that the evidence for appetitive stimuli inhibiting aversively maintained behavior is far less consistent than the reverse, and is highly dependent on the precise temporal relationship between appetitive and aversive events [27,28]. Regardless of implementation quality, when the behavior in question is operantly maintained by a distancing contingency, this approach is more accurately described as a positive reinforcement contingency superimposed on top of an existing negative reinforcement contingency [5,6,29]. This procedural problem has been recognized in the basic behavioral literature: a critical review of animal counterconditioning research concluded that most studies fail to ensure the appetitive stimulus and the aversive stimulus are genuinely paired throughout exposure, since the aversive stimulus frequently disrupts the competing response (e.g., eating) at some point during presentation—meaning the animal is, in practice, often experiencing the aversive stimulus largely unmodified rather than in true simultaneous contact with the appetitive [23].

The problem with superimposition is precisely that it does not address the underlying contingency. Nonlinear Contingency Analysis (NCA) reveals the animal is simultaneously operating under two competing contingencies. A positive reinforcement contingency—for example, food is offered to gain compliance—is layered on top of a negative reinforcement contingency—moving away or displaying aggressive behavior to produce distance. The result is behavioral conflict: approach behaviors are reinforced by food while distancing behaviors are reinforced by removal of the aversive stimulus. These competing contingencies operate simultaneously, and the animal’s behavior reflects both. The behaviors that reflect this conflict are visible and well-recognized by experienced practitioners: the animal takes food while keeping a vigilant eye on the feared stimulus, alternates between approaching for food and retreating, or consumes food with visible tension and truncated normal feeding behaviors [5,6].

Annette Pedersen has described this pattern particularly clearly in the context of the tiger case study presented in this paper, and it is offered here as an illustrative example of a superimposed contingency. The tiger displayed what she termed “conflicted behavior”—consuming food while maintaining fixed vigilance on the person—and the positive reinforcement procedure made no progress toward resolving the underlying distancing contingency because it never contacted the functional reinforcer maintaining that aggressive behavior [5]. As Pedersen observed, this approach was also, in a meaningful sense, punishing the calm behavior: each time the animal settled into food-taking, the aversive person moved closer, making calm behavior the occasion for increased proximity to the very thing the animal was trying to keep away. This same dynamic was independently documented by Squarelli et al. [20]. They describe a comparable conflict in which attempts to introduce food while tigers were still displaying escape or avoidance behavior directed at gaining distance from the trainer produced visible behavioral conflict, and report that aggressive responses were not resolved until distance itself was used as the functional reinforcer, corroborating Pedersen’s account of superimposition failure from an independent research group and species cohort.

When the positive reinforcement contingency is removed—when the feared stimulus is no longer paired with food, or when the food is no longer available—the original undesired distancing behavior is likely to return. This is consistent with experimental evidence demonstrating that when appetitive and aversive contingencies are superimposed, the suppressive effect of the appetitive stimulus on aversively maintained behavior is neither reliable nor durable across conditions [27]. This is not a failure of individual animals or caregivers; it is the predictable consequence of a procedure that addresses the surface behavior without addressing its function when that function is operantly maintained. NCA provides the analytical framework to identify when this is occurring and to redirect intervention toward the functional contingency [2,29]. The time cost of this approach has also been empirically documented: a counterconditioning protocol used to address aversion and escape behavior in a captive capybara (*Hydrochoerus hydrochaeris*) required 25 sessions averaging 2.1 h each—52.5 h of cumulative intervention—before the animal’s fear and escape responses were sufficiently reduced to proceed to subsequent training stages [30]. Associative pairing procedures have legitimate applications elsewhere in animal training—establishing conditioned reinforcers or predictive cues, for example—but we propose they are not well suited to resolving behavior already maintained by an active distancing contingency, as illustrated by the capybara and the tiger case study in this paper.

#### Minimizing Superimposition

In some cases, the physical and social conditions of a training environment make it possible to address a distancing contingency directly, without introducing a second contingency. When an aversive stimulus can be positioned or presented at a distance sufficient for the animal to emit desired calm, oriented behavior, that behavior can be reinforced solely by the controlled removal of the aversive stimulus. Under these conditions, negative reinforcement shaping proceeds without a positive reinforcement contingency present, and the risk of superimposition described above does not arise. The black bear, muskox, and parrot flock cases documented by the authors illustrate this: each animal’s aggressive or fearful response was shaped using distance alone, with food or another appetitive stimulus introduced only later in the procedure when contingencies shifted [4].

The complexity of the zoo environment, however, does not always allow this. Space constraints, the presence of multiple animals, and the practical need to keep an animal positioned and available, or moving, during a session can make a distancing contingency alone impractical. In these circumstances, a positive reinforcement contingency may be introduced for a separate purpose to create a viable starting point for negative reinforcement shaping.

Introducing a positive reinforcement contingency for this purpose carries the same risk of superimposition described above if it is not kept structurally separate from the negative reinforcement contingency operating on the focal behavior. Minimizing this risk begins with treating the two contingencies as governing two distinct behavioral repertoires, maintained by two distinct procedures, at times implemented by two different people. The positive reinforcer—food, for example—is delivered contingent on a behavior unconnected to the animal’s response to the aversive stimulus, such as remaining positioned at a station, rather than being made contingent on tolerating proximity to the aversive stimulus itself.

The second safeguard lies in how the negative reinforcement shaping procedure itself is implemented. Each approximation is selected so that distance is provided before the animal is required to emit a previously effective undesired response, such as escape or aggression, to obtain it. If that response is emitted, it is allowed to produce distance as it always has; this is not treated as a procedural failure but as an indicator that the approximation exceeded what the animal could readily do at that point, and criteria are adjusted accordingly. Maintaining this sensitivity throughout the procedure, rather than pressing for criteria that place the animal in conflict between the two contingencies, is what keeps a positive reinforcement contingency from becoming superimposed on the negative reinforcement contingency it was introduced to support. Both case studies in Section 8 illustrate this structural separation, though implemented differently: the tiger case study stations the animal while the aversive stimulus is presented in approach and retreat, whereas the orangutan case study uses the positive reinforcement contingency itself to move the animal toward and away from the aversive stimulus conditions (night holding).

### 3.3. Misidentification of Procedures in Practice

A persistent source of confusion in zoo animal training is the mislabeling of procedures. In our observation, some practitioners who describe themselves as practicing counterconditioning or systematic desensitization may be engaging in operant shaping, whether with positive or negative reinforcement. While this observation is based on our professional experience rather than formal survey data, it has important implications: the distinction matters because it determines what behavioral mechanisms are engaged and, therefore, what the procedure’s strengths and limitations are.

The key marker is contingency: is the trainer’s behavior contingent on the animal’s behavior? If the trainer moves closer or farther in response to what the animal is doing, the procedure is operant, not exposure-based. If distance is provided (the trainer or stimulus moves away) contingent on the animal emitting a calm response, the procedure involves shaping with negative reinforcement. If an appetitive is delivered contingent on staying calm or for other desired behavior while something aversive is present, positive reinforcement is in play—though it may be superimposed on a negative reinforcement contingency that has not been addressed. Accurately identifying what procedures are in use in an intervention is the necessary first step in evaluating why a given approach is or is not working, and in designing more effective alternatives.

Table 1 provides a comparative overview of the procedures most commonly encountered when addressing behaviors often labeled as fear, aggression, or avoidance in zoo animal training, organized by key procedural features including theoretical basis, functional alignment, contingency contact, role of genuine choice, and welfare implications. Because these procedures may be conflated or mislabeled in practice, the table is designed to support more accurate identification by making explicit what distinguishes each approach at the level of contingency rather than topography. Importantly, the table distinguishes between traditional and constructional applications of both positive and negative reinforcement shaping. This distinction does not imply that functional assessment is absent from traditional applications, but rather that constructional procedures are specifically designed to ensure the selected contingency directly contacts the critical consequence maintaining behavior, preserves genuine choice through maintained degrees of freedom at each approximation, and builds behavioral repertoire rather than suppressing it. The table reflects the paper’s central argument that the welfare profile of a procedure is determined not by the valence of its reinforcing consequence but by these structural features.

## 4. Myths, Misconceptions, and the Controversy Around Negative Reinforcement

### 4.1. The Problem with the Word “Negative”

Perhaps the most pervasive barrier to accurate understanding and appropriate use of negative reinforcement is the word itself. In everyday language, “negative” carries connotations of harm, punishment, and aversion. This has led some animal training communities to conflate negative reinforcement with punishment or with intentional infliction of discomfort. The conflation is compounded by historical applications in which negative reinforcement was used coercively—by inserting aversive stimuli into the environment specifically to evoke escape behavior, as in some approaches to obedience training or livestock management. These applications, while technically instances of negative reinforcement, bear little resemblance to the welfare-oriented, function-based procedures described in this paper.

In behavior analysis, “negative” refers to subtraction: a stimulus is removed or reduced following a behavior, and that removal increases the future likelihood of the behavior [4]. This says nothing about the nature of the stimulus, the practitioner’s intent, or the animal’s welfare experience. Negative reinforcement, used to describe an existing environmental contingency, is simply a description of a functional relationship: the animal’s behavior produces the removal of something, and the behavior increases as a result. Whether this describes a welfare-positive or welfare-negative situation depends entirely on the context and the application. It is important to acknowledge that legitimate concerns about negative reinforcement extend beyond the semantic issue: poorly managed aversive stimuli, insufficient animal control over outcomes, and misreading of behavioral indicators are genuine implementation risks. These concerns are not dismissed here; they are addressed through the structural features of the constructional framework described throughout this paper.

### 4.2. Non-Contrived vs. Contrived Aversive Stimuli

A critical distinction that resolves much of the ethical concern about negative reinforcement is the difference between non-contrived and contrived aversive stimuli. Contrived negative reinforcement involves deliberately inserting an aversive stimulus into the environment to produce escape behavior—for example, applying pressure to cause movement, or using shock to establish a fear response that can then be escaped. This is the form that has historically generated, and continues to deserve, ethical concern.

Non-contrived negative reinforcement, by contrast, involves working with stimuli that are already present in the animal’s environment, or that must be present for legitimate care purposes. The caregiver who is feared by the tiger is not a stimulus inserted to cause distress; the caregiver simply exists in the animal’s world, and the animal’s behavior toward that person is maintained by negative reinforcement. A feared syringe, a microchip reader, a shift door that closes—these are not deliberately inserted aversives but necessary features of care environments. The negative reinforcement procedure works with these existing contingencies rather than creating new ones. This is an important distinction that clarifies the ethical landscape and makes it possible to frame non-contrived negative reinforcement as a procedure that may function as a welfare-positive tool when implemented within a carefully monitored, function-based framework [4].

### 4.3. Myth: Negative Reinforcement Is Inherently Coercive

This myth conflates the existence of an aversive stimulus with coercion. Coercion in behavior analysis is better understood as the restriction of choice: an animal is coerced when it has only one pathway to a critical consequence, and that pathway entails a cost. The antidote to coercion is not the elimination of all contact with aversive stimuli—which would be neither possible nor desirable in most care settings—but rather the expansion of choice through increased degrees of freedom. When an animal has multiple behaviors available that can produce the same functional outcome, degrees of freedom are increased, and coercion is reduced [1,6,8,16].

Negative reinforcement shaping procedures, when implemented within a constructional framework and with genuine choice through multiple degrees of freedom, are specifically designed to minimize coercion and promote choice. Without these principles—without NCA to identify the functional contingency, without degrees of freedom to preserve the animal’s behavioral options, and without the constructional orientation toward building rather than eliminating—negative reinforcement procedures can indeed be coercive. The framework is not an optional refinement; it is what makes the procedure welfare-positive. In practice, this means the animal can always emit the behavior that has historically worked—moving away, vocalizing, or displaying distress—and that behavior will still produce distance, exactly as it always has. What changes is that additional, less costly behaviors are also reinforced with that same distance. The animal is not compelled to emit these new behaviors; they are selected and maintained because they produce the reinforcer more efficiently [6,8,31]. The practical conditions that must be present for meaningful choice to be maintained—including enclosure design, starting distance, behavioral indicator monitoring, and the ongoing effectiveness of distress responses—are addressed in detail in Section 5.3, Section 6.4, Section 7.1 and Section 7.2.

### 4.4. Myth: Using Negative Reinforcement Means Avoiding Positive Reinforcement

The goal of negative reinforcement shaping procedures in zoo animal training is often to create the conditions under which positive reinforcement can be effectively used. When a distancing contingency is in operation, attempts to introduce positive reinforcement are compromised: the animal may respond to appetitives, such as food, while still experiencing the aversive contingency, or may avoid them entirely if the aversive contingency is dominant. By first addressing the distancing contingency—establishing calm or other desired behaviors as functional through negative reinforcement—the practitioner creates a foundation for positive reinforcement to operate without competition. This sequencing is illustrated in the tiger case study in this paper, in which a tiger who chuffed (an affiliative vocalization) in anticipation of a once-feared caregiver’s arrival could then be trained for injections and educational presentations through positive reinforcement [5]. Similarly, the orangutan case study presented in this paper shows that once calm in her night area, she could return to her full repertoire of positive reinforcement-based training. The procedures are sequential and complementary, not competitive [4]. Addressing negative reinforcement contingencies also avoids using deprivation (often of food) to increase the potency of appetitive stimuli in the presence of competing contingencies—a practice that raises its own welfare concerns.

### 4.5. Myth: This Is Too Advanced for Regular Practitioners

Negative reinforcement shaping requires skill—the same skill that positive reinforcement shaping requires. Reading behavioral indicators of calm and distress, accurately timing the delivery of reinforcement, adjusting criteria appropriately, and managing one’s own movement with precision are learned competencies that develop through practice and feedback. These skills are not inherently more difficult than those required for skilled positive reinforcement shaping; they are simply different, requiring attention to different cues and a different timing discipline [4].

The evidence supports the view that applying negative reinforcement within a constructional framework is a learnable, teachable, and accessible skill set. Zoo practitioners across institutions and countries have learned to apply these procedures effectively. Pedersen, describing her team’s experience from a practitioner perspective, noted that the approach “looks funny” at first and “for sure you may feel awkward when you apply it initially, but it certainly works. It absolutely works.” [5]. The learning curve is real, but it is no steeper than that associated with any other nuanced behavioral skill, and the outcomes justify the investment.

Effective application of these procedures requires foundational behavioral knowledge, including the ability to conduct functional assessments, accurately read behavioral indicators of calm and distress, time reinforcement delivery precisely, adjust criteria appropriately, and manage one’s own movement with deliberate awareness. These competencies are addressed in the procedural guidance provided in Section 6 and Section 7 of this paper, and are developed through practice, feedback, and—where possible—supervision by experienced practitioners. They are not beyond the reach of the regular practitioner, but they are not trivial, and the paper does not suggest otherwise.

## 5. Theoretical Framework

### 5.1. The Constructional Approach

The constructional approach, introduced by Goldiamond [1] and further developed by Layng and Andronis [2,7], provides the overarching framework for the procedures described in this paper. Rather than asking how to eliminate a problematic behavior, the constructional approach asks what behavior is absent from the animal’s repertoire that would make the problematic behavior unnecessary—and then sets about building that behavior [1,2].

This reorientation has profound practical consequences. It means that the animal’s current behavior, however challenging, is never the direct target of elimination. Extinction, punishment, and differential reinforcement of incompatible behaviors are not first-line procedures in the constructional approach, because they target the removal of an existing behavior rather than the construction of a new one. Instead, the practitioner identifies the maintaining consequence of the current behavior, uses that consequence to shape new desired responses, and in doing so renders the old behavior functionally unnecessary—which is expected to reduce it over time as the new behavior provides the same functional consequence at lower cost, without requiring its direct suppression [1,2,31].

Five critical elements guide constructional program design [2,4,32]: (1) the terminal repertoire—the ultimate behavioral goal; (2) the current relevant repertoire—the existing behavioral starting point; (3) change procedures—the specific training methods; (4) maintaining consequences—the functional reinforcers that will sustain behavior; and (5) progress monitoring—the metrics for evaluating whether the program is working. These elements apply directly to the design of negative reinforcement shaping programs for distancing-maintained behavior, and are applied explicitly to each case study presented in this paper (see Section 8.1 and Section 8.2).

### 5.2. Nonlinear Contingency Analysis

Nonlinear Contingency Analysis (NCA) is the analytical tool within the constructional approach framework that can help identify the full matrix of contingencies maintaining behavior, including competing contingencies, superimposed contingencies, and the costs and benefits of different behavioral options available to the animal [2]. NCA goes beyond the linear antecedent-behavior-consequence model—which isolates a single behavior and its immediate antecedents and consequences—to examine how multiple contingencies interact and how an intervention might inadvertently activate or ignore some of those contingencies. In applied settings, NCA is not a procedure that automatically reveals what is happening; rather, it is a framework that guides careful observation, hypothesis generation, and evaluation of competing interpretations of behavioral function. Key questions include: What other behaviors could produce the same outcome for this animal? What are the relative costs and benefits of those alternatives? When is the problem not a problem—and what conditions support the desired behavior? What would happen if the animal simply did not engage in the behavior being asked of it? [2].

NCA is particularly valuable in understanding why traditional approaches may be less effective in some cases. When a positive reinforcement contingency is superimposed on a negative reinforcement contingency, NCA can help identify the conflict: two contingencies are competing, and the animal’s behavior will reflect that competition. The caregiver who offers food to a fear-displaying animal is introducing a new contingency—positive reinforcement for approach or staying—that competes with the existing one—negative reinforcement for distance—without altering the latter. NCA helps practitioners identify which contingency is potentiated, what the critical consequence is, and how to design interventions that contact and avoid suppressing the maintaining contingency.

To illustrate how NCA is applied in practice, consider an animal that displays aggressive responses toward a specific caregiver but not others. A linear analysis might focus solely on the aggressive behavior and ask how to reduce it—perhaps by increasing food value or gradually exposing the animal to the caregiver. An NCA approach asks a broader set of questions: What does the aggressive behavior produce for this animal? What other behaviors are available in this context, and what do they produce? Is food-taking occurring alongside vigilance—suggesting two competing contingencies rather than one? When the caregiver withdraws, does the aggressive behavior decrease? Does the behavior occur with all caregivers or only this one, and what does that tell us about the maintaining variable? These questions, taken together, can help identify whether distance from the caregiver is the critical functional consequence—and therefore whether a procedure that directly contacts that consequence, rather than overlaying a new one, is indicated. The case studies presented in Section 8 illustrate this process in full detail.

### 5.3. Degrees of Freedom, Genuine Choice, and Assent

Goldiamond’s concept of degrees of freedom (n − 1, where n is the number of behavioral pathways to a critical consequence) provides a precise technical framework for understanding and promoting genuine choice [1,8,31,33]. As used here, degrees of freedom does not refer simply to the existence of multiple behavioral options, but to the specific condition in which two or more behaviors can produce the same functional consequence—meaning the animal is never required to emit any single specific behavior to access what it needs. An animal with only one pathway to a critical consequence has zero degrees of freedom and is, in that sense, coerced. An animal with two or more functional pathways has at least one degree of freedom and experiences meaningful choice in the technical sense intended by this framework. Readers seeking a fuller treatment of this concept and its theoretical foundations are directed to Goldiamond [1,8], de Fernandes and Dittrich [31], and Linnehan et al. [34].

We acknowledge that real management conditions—enclosure design, keeper proximity, shift door constraints, time pressure, and safety requirements—influence how degrees of freedom are structured in practice. These factors shape intervention design and require thoughtful procedural planning, but they do not preclude the inclusion of degrees of freedom in the procedure. Creative problem-solving within management constraints is precisely what constructional program design asks of practitioners, and the case studies in Section 8 illustrate this directly: in the tiger case, enclosure geometry required redesigning the starting conditions to establish sufficient distance, and in the orangutan case, the procedure was implemented under genuine time pressure while still preserving the animal’s ability to retreat and reset at every approximation. The minimum conditions necessary for degrees of freedom to be genuinely present in negative reinforcement shaping procedures are addressed in Section 6.4 and Section 7.2, which describe the environmental and procedural requirements that must be in place before and throughout the procedure.

In constructional programs using negative reinforcement shaping procedures, degrees of freedom are built into the procedure by design from the outset. The animal can always emit the behavior that has historically worked—moving away, vocalizing, displaying distress—and will still receive the distance that behavior has always produced. This ensures the animal is never asked to give up access to its critical consequence. What changes is that additional behaviors are also reinforced with that same consequence, giving the animal more ways to achieve what it needs and therefore greater behavioral freedom [6,34].

Assent, in this context, refers to the animal’s ongoing engagement with the interaction under conditions where functional alternatives—including disengagement or distancing—remain genuinely available and effective, rather than engagement occurring because no other option exists [35]. Assent is not a single, fixed event but an ongoing process, evident in the animal’s behavior throughout every session. Critically, behavioral topography alone cannot confirm assent: an animal may display calm or compliant behavior while still operating under a dominant aversive contingency, as the superimposition problem discussed in Section 3.2 illustrates. Genuine assent is indicated by the animal’s continued engagement when functional alternatives—including the option to disengage or produce distance—remain available and effective. It is the presence of those functional alternatives, not the appearance of any particular behavior, that makes assent meaningful. The full operational treatment of assent as a behavioral and ethical construct is available in Abdel-Jalil et al. [35] and Linnehan et al. [34], to which readers are directed for the deeper discussion that this important topic warrants but that falls outside the primary scope of this paper.

## 6. Assessment: Identifying Distancing Contingencies and Planning for Intervention

### 6.1. Step One: Is the Behavior Maintained by Distancing?

The first and most important question before implementing a negative reinforcement shaping procedure is whether the behavior in question is maintained by a distancing contingency—that is, whether the animal’s behavior functions to produce distance, removal, or escape from something, and whether that outcome is what keeps the behavior occurring. This is a functional question, not a topographical one. Two animals displaying very different-looking behaviors—one growling, one freezing—may both be operating under a distancing contingency. What matters is what the behavior produces, not what it looks like.

While topography cannot by itself establish function, certain observable features can help practitioners generate a hypothesis about which contingency is operating—a hypothesis then confirmed by testing what happens to the behavior when the consequence changes. Useful observational questions include: Does the animal orient away from a stimulus, or attempt to drive it away? Does the behavior decrease or stop when the aversive stimulus moves away? Does emotional behavior appear loose, fluid, and exploratory, or tense, vigilant, and guarded? Does engagement with appetitives decrease in the presence of a particular stimulus and return when that stimulus moves away? These observations help distinguish distancing contingencies—maintained by negative reinforcement—from nearing contingencies—maintained by positive reinforcement—and point to which procedure is functionally indicated.

If the assessment indicates a distancing contingency is operating, a negative reinforcement shaping procedure is indicated, though practical implementation factors including enclosure design, staff skill, animal health status, and safety considerations should also inform that decision. The competencies required for effective implementation are addressed in Section 4.5, and practitioners seeking more structured assessment guidance are directed to the constructional questionnaire in Layng et al. [2].

### 6.2. Step Two: Identify the Specific Aversive Stimulus or Conditions

Once it has been established that a distancing contingency is operating, the next task is to identify precisely what the animal’s behavior is functioning to escape. This requires careful observation rather than assumption. Common categories of aversive stimuli in zoo settings include people (specific individuals, strangers, groups, or people in particular contexts), objects (medical equipment, tools, novel items), locations or spaces (shift areas, chutes, night houses, transport containers), events (door movements, noise, touch, approach), other animals, or combinations of these. Aversive conditions may also be multi-factorial: avoidance may be influenced by a combination of person, location, sound, door movement, social context, or prior experience operating together, and targeting only one element may not fully resolve the behavior if multiple aversive conditions are contributing.

It is important to note that the same stimulus may function as an aversive under some conditions but not others. A caregiver with an established, non-aversive interaction history in one context may function as an aversive when wearing unfamiliar equipment or working in an unfamiliar space. A shift door that has been used without incident for years may become aversive after a single frightening event. The practitioner’s task is not to find a general label for the stimulus but to identify the specific context in which it occasions the distancing behavior. Identifying this as precisely as possible is essential, because the intervention will use that specific stimulus or stimulus conditions—and the animal’s ability to gain distance from it—as the functional reinforcer.

### 6.3. Step Three: Check for Competing Contingencies

A distancing contingency does not always operate in isolation. Consistent with NCA [2], the practitioner checks whether a positive reinforcement contingency is also present in the same context, and whether a prior intervention has already superimposed a contingency that was never resolved (see Section Minimizing Superimposition). This is particularly important when food or another appetitive has previously been used in the presence of the aversive stimulus, since superimposition may already be in place without having been identified as such. Signs include conflicted behavior—the animal engaging with an appetitive while also displaying distancing responses toward the aversive stimulus—or a prior intervention history that relied on pairing food with proximity to the stimulus. Identifying this before beginning shaping allows the intervention to contact the critical consequence directly, rather than layering a new contingency on top of one that remains unresolved.

### 6.4. Setting up for Success: The Training Environment

An important part of shaping with negative reinforcement is creating conditions in which the animal can readily emit desired responses, so those responses can be reinforced by the animal gaining distance from the aversive stimulus. The training environment must therefore be assessed before the first session to determine whether the conditions support this goal.

Key environmental considerations include whether the aversive stimulus can be removed, modified, or introduced gradually; whether there is sufficient space for the animal to get enough distance from the aversive stimulus to emit calm behavioral indicators; and how first contact between the animal and the stimulus will be arranged—whether the stimulus will approach the animal, the animal will approach the stimulus, or another behavior (such as a stationing behavior or eating while the stimulus is present at a distance) will be used to facilitate the first approximations.

Sufficient starting distance is the most critical element. “Sufficient” means the animal is displaying desired behavioral indicators such as calm at the outset: relaxed muscle tone, normal posturing, absence of alarm vocalizations, and engaging with the environment in typical ways. If the aversive stimulus cannot be placed at a sufficient distance to allow desired responses at the outset—as occurred in the first attempt at the tiger case discussed below, where the enclosure geometry made adequate distance difficult—the intervention must be redesigned before proceeding [5,36].

## 7. Applying Negative Reinforcement Procedures: Principles and Nuances

### 7.1. Reinforcing Desired Behavior: What to Look for and When to Reinforce

In negative reinforcement shaping, the reinforcer is the removal or reduction in the aversive stimulus or stimulus conditions. This means the animal or stimulus moves away, or the stimulus conditions are reduced in some other way (e.g., by turning away, increasing distance, or reducing salience), contingent on the animal emitting desired behavioral responses. Timing is critical: distance should be provided promptly when calm or desired behavior is observed, just as the delivery of appetitives in positive reinforcement shaping should occur promptly when the desired behavior is emitted [4,5].

In constructional negative reinforcement shaping programs, multiple behaviors can and should be reinforced with distance—this is how degrees of freedom are built and how rapid behavior change is facilitated. Rather than reinforcing only one specific behavior, the practitioner reinforces a wide range of behavioral indicators of calm or other desired responses. These may include: attending to the stimulus and staying in position; increased time in contact with the stimulus without behavioral changes; decreased distance from the stimulus without behavioral changes; relaxation indicators such as softening of eye shape, reduced muscle tension, weight dispersal shifts, relaxed tail, or resumption of eating; self-care behaviors such as grooming, scratching, or head shaking; orientation to the environment, including attention to conspecifics, objects, or sounds not related to the aversive stimulus; calm movement in the direction of the stimulus, whether directed (e.g., the animal orienting toward or approaching the stimulus with gaze directed at it, as if to investigate) or non-directed (e.g., movement that produces proximity as a byproduct of other behavior not directed at the stimulus); and eventually affiliative responses such as approaching, sniffing, or eating food with a calm posture in the presence of the stimulus. These indicators should be operationally defined for the individual species and individual animal before implementation, as the behavioral expression of calm, hesitation, and distress varies across taxa and individuals.

This range of reinforceable responses is important for several reasons. First, it may increase the speed of behavior change by keeping reinforcement frequent and contingent on small, readily emitted approximations. Second, it builds a broad repertoire of behaviors that produce the desired outcome, increasing degrees of freedom and reducing coercion. Third, it provides the practitioner with a rich source of behavioral information: which response options an animal emits when reinforcement is available across multiple alternatives reveals which approximations are well-established and which remain low-probability or effortful.

### 7.2. Preserving Degrees of Freedom: Letting Distress Responses Work

A defining feature of negative reinforcement shaping done in alignment with constructional principles is that the animal’s distress responses—the very behaviors the intervention is designed to address—are never extinguished. If the animal displays elevated fear or aggression, the stimulus moves away. Distance is still delivered. The program resets to the last successful criterion and proceeds from there.

This is not a concession or a procedural failure; it is a principled commitment to the animal’s agency. By ensuring that distress responses continue to work—that the animal never loses access to its critical consequence—the procedure maintains at least one degree of freedom at all times. The animal always has two ways to get distance: emitting calm or other identified desired responses or displaying distress. Over time, desired responding becomes more efficient and less costly than distress responding, and the animal’s behavior shifts accordingly. This shift is not coerced; it is selected because desired behaviors work better [1,6,26,31,34].

This approach stands in direct contrast to extinction-based procedures, in which previously effective behaviors are rendered ineffective. The frustration and aggression that often accompany extinction—well-documented in the behavioral literature and observable in zoo settings—are not expected in negative reinforcement shaping done as described in constructional programs, as the mechanism that produces them—rendering previously effective behavior ineffective—is specifically avoided [2,6,7].

### 7.3. The Switchover: Recognizing and Responding to Behavior Change

Practitioners familiar with these constructional procedures describe a characteristic “switchover” moment, first described by Rosales-Ruiz and his students [9,10,12], a point at which the animal’s behavioral presentation changes from watchful avoidance or active distress to something more like curiosity and active engagement. The animal may begin orienting toward the previously aversive stimulus rather than away from it, approaching rather than retreating, or showing interest—ears forward, relaxed posture, investigatory behavior—rather than tension. This shift may indicate that the functional relationship between the stimulus and the animal’s behavior has changed [5].

The switchover is not always dramatic or instantaneous, but it is often recognizable and frequently occurs more rapidly than practitioners expect. It signals that reinforcement history for new behaviors has been established and that the distancing contingency is no longer the dominant maintaining contingency. At this point, appetitive stimuli can function as effective reinforcers without competing with the distancing contingency, and food or other appetitives can be cautiously assessed and introduced, beginning with small amounts at sufficient distance to avoid reintroducing a competing contingency, following the same safeguards against superimposition described in Section Minimizing Superimposition [5,26].

## 8. Case Studies

### 8.1. Case Study One: Modifying Aggressive Responses Toward a Specific Caregiver in a Male Amur Tiger (Panthera tigris altaica), Copenhagen Zoo

#### 8.1.1. Case History

The subject was a male Amur tiger who arrived at Copenhagen Zoo in March 2022. As the authors have commonly observed for large felids relocated to a new facility, he required a period of habituation to his new environment and to the staff who would be caring for him. Most caregivers used a combination of familiar management procedures and repeated routine contact to establish a working relationship, and he eventually established calm, affiliative responding with all but one member of the animal care team. That individual, referred to here as Holger, was a skilled and experienced animal caregiver for whom the tiger displayed persistent aggressive responses: growling, staring, raised lip behavior, and sustained vigilance, even when Holger was not in direct proximity. The tiger would growl at the sound of Holger’s voice even before seeing him, suggesting that auditory stimuli associated with Holger had acquired aversive properties.

The differential nature of the tiger’s response, calm with all other caregivers but aggressive toward this specific individual, was inconsistent with generalized fear of people or novelty as the maintaining variable and pointed to the specific associative history between this tiger and this caregiver as the relevant contingency. The cause was never conclusively identified; no apparent difference in Holger’s appearance, voice, or behavior could be isolated as the precipitating factor. The aggressive responses were consistent across all interactions with this caregiver: they occurred each time Holger was present, persisted for the duration of his presence, and showed no variation across days or contextual conditions. For the purposes of this case description, the behaviors are operationally defined as follows: growling refers to audible low-frequency vocalizations directed toward Holger; staring refers to sustained forward-directed gaze maintained across his approach; raised lip behavior refers to retraction of the upper lip exposing the canines; and sustained vigilance refers to maintained oriented body posture and gaze toward Holger regardless of other ongoing activities.

#### 8.1.2. Functional Assessment

Before the negative reinforcement procedure was implemented, Zaco, the senior caregiver, had applied what the team characterized as systematic desensitization and counterconditioning, using the approach most familiar to the team: he sat quietly near the tiger’s space, allowed the tiger time to observe him, gradually introduced food when the animal showed reduced tension, and allowed Holger to move progressively closer to the tiger while another caregiver provided preferred food items. This approach had worked for all other caregivers. With Holger, it did not. Precise session counts and progression criteria from the earlier desensitization attempts were not recorded, as the intervention predated any plan to document this case for publication; this represents a limitation of the retrospective case format.

Pedersen’s analysis of why it failed is instructive and worth examining in detail. The procedure relied on the extinction of aggressive behavior: the tiger was expected to cease displaying aggressive behavior as Holger drew progressively closer. Simultaneously, food was offered to create an appetitive association with Holger’s presence, the counterconditioning component. What was observed was visible conflict: the tiger consumed food while maintaining fixed vigilance on Holger, and the team could observe that it “wanted food, but clearly wanted the person to leave.” [5,36]. Observable behavioral indicators of this conflict included interruption of food intake when Holger moved closer, resumption of food-taking when he withdrew, and sustained body orientation toward Holger throughout food consumption. Every time Holger moved closer and the animal remained calm enough to eat, it was being exposed to more of the aversive stimulus, not less, without the behavior that had historically worked (aggressive behavior producing distance) being effective. Contingency analysis suggests that calm food-taking was, in effect, being punished by progressively closer proximity to the aversive stimulus.

This analysis illustrates the core problem of superimposition: the positive reinforcement contingency (food for staying) was layered on top of the negative reinforcement contingency (aggressive behavior for distance) without addressing the latter. The tiger’s distress and conflict behaviors were not signs of stubbornness or intractability; they were the predictable behavioral signature of competing contingencies.

#### 8.1.3. The Negative Reinforcement Shaping Procedure

The negative reinforcement shaping procedure was designed to help contact the contingency maintaining the aggressive behavior: the tiger’s aggressive behavior produced distance from Holger, and this was the functional reinforcer maintaining the behavior. The procedure would use this same reinforcer, distance, to shape calm behaviors instead.

Setup: All training was conducted via protected contact. Zaco, Holger, Pedersen, and other participating team members worked from the designated animal care staff area, while the tiger remained secure in his inside holding area. Zaco was present in the animal care staff area close to the tiger, providing food contingent on the tiger remaining settled—reinforcing lying down and calm, positioned presence—rather than on any response to Holger. This gave the tiger a behavioral reason to remain in the space and provided a stable starting point—relaxed posture, engagement with food, and absence of vigilance responses—from which the negative reinforcement shaping with Holger could begin. Zaco could not observe Holger’s position or movements directly, and the food was not connected to Holger’s approach. This kept the two contingencies operating on separate behaviors, consistent with the safeguards against superimposition described in Section Minimizing Superimposition: food access depended on a behavior unconnected to Holger’s approach, while distance depended solely on the tiger’s response to Holger. Throughout the procedure, the tiger retained the option to use previously effective behaviors—growling, staring, or other aggressive responses—to produce distance; these behaviors were never made unavailable. Approximations were kept small enough that the desired response could be reinforced before the established behaviors were occasioned, which was the primary mechanism for minimizing the risk of superimposition of the two contingencies [36].

Procedure: Holger, coached by Pedersen and other team members positioned in the animal care staff area where they could observe both Holger and the tiger, began at a distance of approximately two meters—the minimum distance permitted by the structural design of the building—where the tiger could observe him without displaying aggressive responses. He then stepped briefly forward, a small increment, and immediately stepped back before the tiger displayed aggressive responses. Distance was the reinforcer: the brief approach followed by withdrawal reinforced calm, oriented behavior toward Holger—soft body posture, quiet vocalization, and relaxed engagement.

Holger watched the tiger’s eyes and body posture closely, particularly facial tension indicators (whisker position, lip tension, ear set), as signals of when to advance and when to retreat. The timing had to be precise: stepping back too late occasioned the tiger to escalate into a more aggressive response, reinforcing that response with distance rather than a calmer approximation; stepping back too soon meant withdrawal occurred before any desired response had been emitted, leaving no approximation to build on. When a low-level aggressive indicator (whisker rise, lip tension) appeared, Holger withdrew immediately, reinforcing even this low-level response with distance and preventing escalation.

When, in one session, the tiger did growl at Holger, the response was immediate withdrawal rather than an attempt to wait out the behavior or hold at that criterion. Allowing the growl to produce distance, rather than withholding it, meant the previously effective behavior remained available, and the tiger was not placed in conflict between the two contingencies operating in the session—the outcome Section Minimizing Superimposition identifies as the mechanism for reducing the risk of superimposition, rather than an incidental result of small approximations alone. As Pedersen explained to her team: “If we by accident make him growl and stay too long for him to cope, we would rather reinforce a very low degree of aggressive response instead of letting it escalate.”

#### 8.1.4. Outcomes

The results of this procedure were documented in video and in the training logbook with session-by-session behavioral observations. The shaping phase comprised eight sessions conducted over approximately five weeks, totaling roughly 42 min of direct contact time—a notably brief investment given the severity and persistence of the prior aggressive responding. Sessions ranged from approximately 3.5 to 8 min in duration and were spaced across days based on animal and staff availability. Progress across sessions reflected the gradual shaping process: early sessions established that Holger could be present at a distance without occasioning aggressive responses; subsequent sessions incrementally reduced distance and increased the duration of Holger’s presence as the tiger’s behavioral indicators supported advancement. In one session, complications arose when a female tiger in an adjacent area was entering estrus, which increased the male’s arousal, potentiating aggressive responses; the team responded by reducing criterion demands rather than pressing forward, consistent with the procedural logic of reinforcing the lowest effective criterion and avoiding escalation. The switchover was observed in Session 8, when the tiger showed no aggressive response or vigilance—no sustained, oriented body posture or directed gaze—toward Holger, in response to Holger standing directly at the fence for ten seconds—a criterion that had previously reliably occasioned aggressive behavior. Three consolidation sessions followed, comprising approximately 20 additional minutes of contact, during which the team verified the behavioral change across increasing proximity and duration before transitioning to joint hand-feeding and ultimately to Holger feeding and training the tiger independently. These consolidation sessions were not a continuation of the shaping procedure but a deliberate verification phase—the team did not assume permanence from the switchover alone [36].

The most significant marker of success was what Pedersen observed as “the switchover”: the point at which the tiger stopped monitoring Holger with suspicion and began to show what appeared to be curiosity or anticipation. He began to chuff, a low-frequency affiliative vocalization typical of tigers, when he heard Holger approaching. This is reported as one behavioral indicator among several marking the shift in the tiger’s responding, and was observed by the care team as a notable change in an animal they had believed might never tolerate Holger’s presence.

Following the resolution of the distancing contingency, Holger was able to transition the tiger to positive reinforcement training. Holger subsequently took a lead role in the tiger’s training, conducting medical procedures and eventually participating in educational presentations with guests, interactions that would have been impossible before the intervention. No regression to aggressive responding has been observed since the switchover in March 2023. Holger has continued to work with this tiger on a regular basis and remains an active member of the training team.

#### 8.1.5. Procedural Notes and Welfare Considerations

Several features of this case are worth highlighting for practitioners:

- Distance as the reinforcer. The identification of distance from Holger as the critical consequence—the outcome the tiger’s aggressive behavior had always produced—was the foundation of the intervention. Structurally separating the two contingencies, food managed by a second caregiver and contingent on a behavior unrelated to Holger’s approach, was the first of the two superimposition safeguards described in Section Minimizing Superimposition.
- Timing precision as a welfare consideration. The procedure required Holger to identify subtle behavioral indicators—whisker position, lip tension, ear set, and eye contact—as signals of when to advance and when to withdraw. Stepping back too late risked allowing the tiger to escalate the aggressive response; stepping back too soon meant the approach had not selected behaviors from the desired repertoire. In this case, that level of observational precision benefited from direct coaching by Pedersen and real-time feedback from team members positioned to observe both Holger and the tiger simultaneously, suggesting that video review and hands-on coaching may support skill development in this area.
- Responding to low-level aggressive responses. When the tiger growled in one session, Holger withdrew immediately rather than waiting for the behavior to subside. Reinforcing even a low-level aggressive response with distance was the deliberate choice—treated as an indicator that the approximation had exceeded what the tiger could readily do, not as a procedural failure. This is the second of the two superimposition safeguards described in Section Minimizing Superimposition: previously effective behaviors remained available throughout, thereby minimizing the likelihood that the tiger would be placed in conflict between the two contingencies.
- Adjusting criterion to environmental context. In one session, the presence of a female tiger in an adjacent area entering estrus appeared to elevate the male’s arousal. The team responded by reducing criterion demands for that session rather than pressing forward. This flexibility is consistent with the constructional logic of reinforcing the lowest effective criterion and reflects one approach to welfare-sensitive practice under variable environmental conditions.
- Limitations of this case report. The negative reinforcement procedure was prospectively documented through video and a training logbook. The prior systematic desensitization phase was not recorded with equivalent detail, as it predated any plan to publish this case; precise session counts and progression criteria from that earlier intervention are therefore unavailable. This case is presented as a practitioner case report rather than a controlled study and should be interpreted accordingly.

#### 8.1.6. Analysis Through the Constructional Approach Framework

Table 2 summarizes the application of the five critical elements of the constructional approach to this case, providing a structured overview of how the theoretical framework guided each stage of the intervention.

Viewed through the five elements of the constructional approach [1,2,32], this case illustrates each component in practice. The terminal repertoire was calm, affiliative interaction between the tiger and Holger sufficient to support medical training. The current relevant repertoire included the tiger’s already-functional capacity to regulate distance—a competent behavioral tool the animal was actively using to manage its environment—along with any existing moments of calm or neutral orientation toward Holger that could serve as a constructional starting point. Rather than treating distancing behavior as a pathology to eliminate, the program recognized it as a meaningful and usable behavior, indicating what consequence was critical. The change procedure contacted that critical consequence, using distance itself as the functional reinforcer to shape progressively calmer behavioral responses. The maintaining consequences shifted across the program—initially distance, subsequently food and social engagement. This shift was not assumed but confirmed behaviorally: the absence of aggressive or vigilant responding under a criterion that had previously reliably occasioned them indicated the distancing contingency was no longer dominant, supporting the transition to positive reinforcement as the tiger’s repertoire expanded to access reinforcement through affiliative rather than avoidance behavior. Progress monitoring was embedded in the videos, the training log, and the documented behavioral indicators across sessions, enabling ongoing evaluation of whether the program was moving toward the terminal repertoire.

The degrees of freedom present throughout the procedure meant the tiger could always emit aggressive behavior and receive distance, ensuring that the animal was never required to give up access to its critical consequence. What changed was that additional, less costly behaviors also produced that consequence, making the aggressive behavior functionally less necessary. Its frequency decreased not because it was extinguished but because, consistent with an NCA-based functional account, it was no longer the most effective or necessary pathway to what the tiger needed.

### 8.2. Case Study Two: Addressing Newly Acquired Avoidance of a Night Holding Area in an Orangutan (Pongo pygmaeus), Dublin Zoo

#### 8.2.1. Case History

The subject of this case study was a female Bornean orangutan (*Pongo pygmaeus*) residing at Dublin Zoo. The orangutan had an established behavioral repertoire and was cooperative, working readily with all members of the animal care team. She was notably socially responsive, showing particular affinity for a younger male orangutan who had joined the group in late 2025. She was described by the animal care team as eager to engage in training, responsive to caregivers, and generally a reliable participant in behavioral husbandry. Shifting reliably between areas on cue was a well-established behavior; prior to this incident, she had shifted consistently and without hesitation with all members of the care team.

The incident that gave rise to this case study occurred during Heidenreich’s consulting visit in April 2026. On one evening of the visit, a planned late-night power outage unexpectedly triggered the building’s fire alarm, which sounded loudly and for an extended period. The outage also caused ventilation panels to open, allowing cold air to enter the indoor holding area overnight. It is reasonable to hypothesize that this combination of stimuli—sudden, loud, and sustained alarm sounds; cold air temperature; and the disruption of the normal evening environment—constituted an aversive experience for the animals, and, based on behavioral observations, the one female in particular appeared most affected.

The following morning, when staff members arrived, the two animals appeared calm but less rested and were shifted to the other side of their indoor habitat after a separately housed female in the group moved to the outdoor habitats for the day. This was a sensible management decision that gave them access to a space without an aversive history associated with the previous night’s events. The animals behaved normally throughout the day. In the afternoon, when the team attempted to shift the animals back to prepare for the other female to come off the outdoor habitats, the female in question was visibly hesitant to return to the area where she had spent the night during the power outage, despite conditions having been restored to normal. The male had already shifted and was comfortably nesting.

This hesitation was not trivial. The other female orangutan in the group, who was waiting outside, needed to shift into the area currently occupied by this female, and the operational management of the group required that the female currently inside shift back into her night-holding area and that the shift door close behind her. The situation was time-sensitive, and the avoidance behavior was hypothesized to be maintained by the aversive history associated with that space from the previous night.

#### 8.2.2. Functional Assessment

The functional assessment was based on the observed abrupt change in behavior following a specific environmental event. The orangutan had a consistent and reliable history of entering the night holding area prior to that night. During the night in question, she experienced loud sounds, cold temperatures, and disrupted sleep while in that space. Following this event, she began to display hesitation and avoidance at the doorways leading to the night holding area—behavioral indicators that the space had acquired aversive properties, likely through its association with the prior night’s conditions. Based on our hypothesis and assessment, the critical consequence was distance from the night holding area; hesitation to enter was maintained by negative reinforcement, with access to the preferred main house serving as the context in which that distance was available.

An additional consideration identified by the care team was a new sensitivity to the shift door closing behind her, absent prior to the incident. The team considered it possible that closure had acquired aversive properties as part of the space’s broader association with the previous night’s events, given that closure reliably restricted her access to the main house.

#### 8.2.3. The Negative Reinforcement Shaping Procedure

A note on the two-contingency structure of this procedure is warranted before describing the mechanics. The aversive stimulus conditions in this case—the night holding area—could not be moved or removed; the orangutan had to be shaped to approach and enter it. This required two distinct contingencies operating on two distinct behaviors. First, movement into the den spaces needed to be occasioned and reinforced: caregiver positioning, body orientation, verbal cues, hand gestures, and the visibility of food directed the orangutan toward successive approximation points, with food delivered contingent on arrival at each point. Second, and separately, calm behavioral indicators while in the aversive space needed to be reinforced: the opportunity to return to the main house—distance from the night house—served as the critical consequence for that behavioral repertoire, building reinforcement history for calm, approach-oriented responses in that context with minimal exposure to the aversive stimulus conditions. Consistent with the first safeguard against superimposition described in Section Minimizing Superimposition, these two contingencies were deliberately kept on separate behaviors: food reinforced movement and arrival; distance reinforced calm behavioral repertoires. Together, they made it possible to move the animal toward and away from the stimulus conditions in a carefully shaped progression.

**Figure 1:**
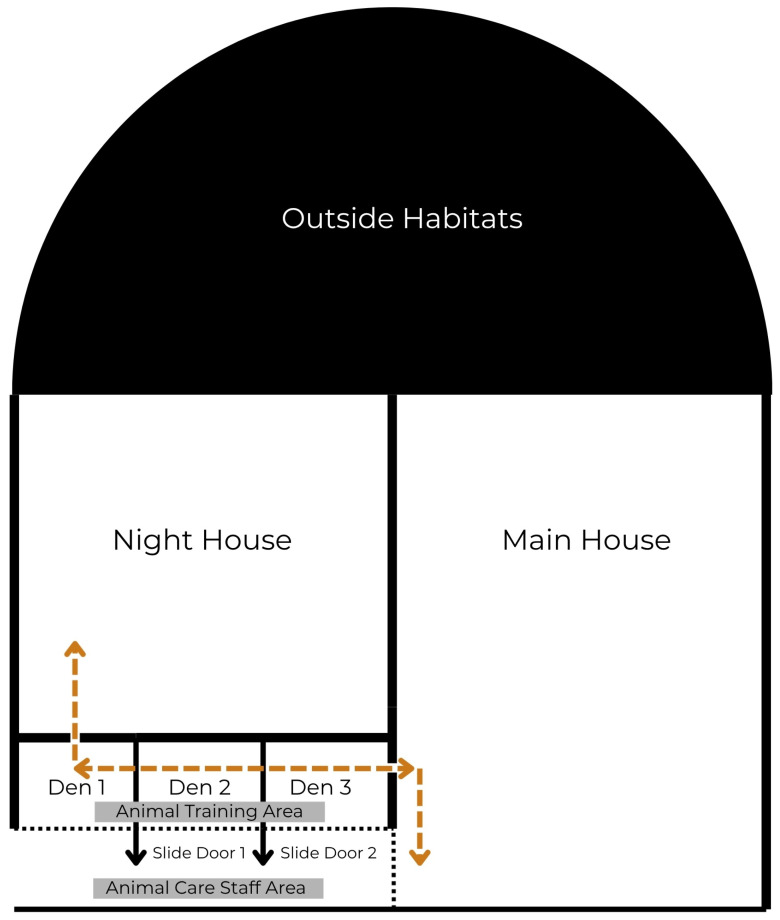
Schematic layout of the orangutan facility at Dublin Zoo. All three dens serve as animal training areas where staff work with the orangutans in protected contact. The orangutan began in the Main House and was cued to make successive approximations progressively deeper into the den areas—first into Den 3, then Den 2, then Den 1—with return to the Main House serving as the reinforcer after each approximation. Orange bidirectional arrows indicate this back-and-forth movement of the orangutan. Black arrows indicate slide doors. Once the orangutan had entered the night house and her behavioral indicators were calm, the sliding shift door between Den 2 and Den 3 was manually closed.

The procedure proceeded as follows. The orangutan was cued by team members in the animal care staff area to move toward and partially into Den 3 (Figure 1)—a passageway leading toward the night house—using the caregiver cues described above. Food was delivered contingent on arrival at each approximation point, both upon entry into the den spaces and upon return to the main house, reinforcing movement in both directions. The team monitored her behavioral indicators carefully throughout—posture, movement quality, sitting in doorways, hanging onto mesh, reaching with arms, leaning, darting looks for escape paths, orienting behavior—as signs that the approximation had gone too far or that it was time to return. Consistent with the second safeguard described in Section Minimizing Superimposition, if the orangutan displayed one of these indicators or attempted to retreat, that response was allowed to produce distance rather than being withheld or waited out; retreat remained available throughout and was treated as information that the approximation exceeded what she could currently do, not as a procedural failure. They deliberately avoided closing any shift doors during the initial approximations, recognizing that door movement itself could constitute an additional aversive event before the orangutan had established sufficient comfort with the space.

As reinforcement history was built for more desired responses, the criteria were gradually extended: slightly deeper entry into the den, slightly longer duration before being signaled to return, sitting on bench stations, arms relaxed by her body, more orientation toward caregivers, and less vigilance—defined here as reduced scanning toward the main house, decreased orientation toward escape routes, and absence of reaching through doorways or leaning away from the space. Progress toward the terminal repertoire was reflected in the orangutan sitting fully within the den rather than in a doorway, orienting toward caregivers rather than toward exits, and remaining at her station on the wooden platform in Den 2 for increasing durations. The team accepted durations of approximately 10 to 30 s of calm, settled presence as meaningful progress; in early approximations, even a single second of contact—approaching, taking food, and returning immediately to the main house—represented the available criterion. A notable shift occurred when the orangutan began remaining at her station to eat and wait for more food rather than retreating immediately (an indicator of possible superimposition), and the team found themselves needing to cue her to leave rather than waiting for her to do so spontaneously. As with the switchover described in the tiger case (Section 8.1.4), this suggested food was beginning to function as a reinforcer without competing against an unresolved distancing contingency. Rather than treating this as license to extend criteria, the team continued to cue her to return before any fear response could be occasioned, consistent with confirming rather than assuming behavioral change.

Prior to the incident, closing Slide Door 2 (Figure 1) while the orangutan was stationed in Den 2 had been a routine component of the shift procedure that occasioned no avoidance responses. However, during this session, a small movement of the door handle occasioned an escape response, and she retreated to the main house—consistent with the safeguard preserving her ability to use previously effective escape behavior. She quickly returned to her station, though, and the team was able to build upon this approximation until she entered the night house completely. When the orangutan entered the night area and her behavioral indicators remained calm, the team judged these to be sufficient to attempt to close Slide Door 2 again and was able to do so without incident. At this point she calmly approached the male and engaged in social interactions. The team’s communication was deliberate and calm throughout; although time pressure existed, the team deliberately slowed down to thoughtfully implement the procedure, attending to the orangutan’s behavioral indicators at each step rather than pressing toward the management goal. The other female orangutan shifted into the freed-up space shortly thereafter. Behaviors for all three animals were back to their typical responses by the same time the following day.

#### 8.2.4. Outcomes

The shifting procedure took approximately 15 min from start to finish as evidenced by video data. Within that window, the female orangutan progressed from refusing to enter the night-holding area to being fully shifted into it, with the door closed behind her, demonstrating calm behavioral indicators throughout the final phases of the process. The management goal was accomplished; the other female could be shifted into the space vacated by the female whose behavior had been quickly shaped, with calm behavioral indicators maintained throughout the final phases—relaxed posture, oriented engagement with caregivers, and absence of escape responses—and without any observable deterioration in the animal’s training relationship with the care team, as evidenced by her return to typical cooperative responding the following day and maintained since.

This outcome was particularly notable given the time pressure under which it was achieved and the fact that the aversive history was recent and apparently salient for the orangutan. The negative reinforcement procedure was not implemented because it was the theoretically preferred option after extended deliberation; it was implemented because the team recognized in the moment that this was the appropriate functional response to the behavioral situation they were observing. This case illustrates how coordinated team communication and rapid functional assessment may support welfare-sensitive management under time pressure.

#### 8.2.5. Procedural Notes and Welfare Considerations

Several features of this case are worth highlighting for practitioners:

- Distance from the night house was the reinforcer. Two separate contingencies structured the procedure; however, distance from the night house—the outcome her refusal to enter had always produced—was the critical consequence, and identifying it was the foundation of the intervention. Food, delivered by caregivers, reinforced movement toward and arrival at approximation points, while distance reinforced calm behavioral repertoires within the aversive space. Keeping these two contingencies on separate behaviors was the first of the two superimposition safeguards described in Section Minimizing Superimposition. The second was preserved throughout: when the orangutan displayed retreat indicators, including the escape response occasioned by the door handle movement, that response was allowed to produce distance rather than being withheld, keeping previously effective escape behavior available and minimizing conflict between the two contingencies.
- Door management as a welfare consideration. The team’s deliberate attention to Slide Door 2—holding it open or moving it only in predictable, non-surprising ways throughout the procedure, and treating its closure as its own final approximation rather than a byproduct of getting the orangutan into the space—exemplifies how welfare-sensitive procedural refinement looks in practice. A single poorly timed door movement could have escalated her distress and reset her progress; closing it prematurely, before her behavioral indicators were ready, would have created an additional aversive pairing.
- The role of the team. The procedure succeeded in part because multiple team members were present, communication was clear, and roles were understood. Managing large great apes in complex environments requires coordinated team responses, and the success of this intervention reflects the team’s behavioral fluency. Specifically, one caregiver cued the animal and monitored behavioral indicators from inside the animal care staff area, a second caregiver managed door movement, and Heidenreich observed and communicated progression and reset decisions to the team, with additional team members supporting management of the broader animal group and habitat access throughout.
- Limitations of this case report. This was a time-sensitive management situation that arose without warning and was not prospectively designed as a study. The procedural detail reported here reflects what was observable and documented in real time, supplemented by video review. It is presented as an applied case report rather than as controlled evidence of general effectiveness, and should be interpreted accordingly.

#### 8.2.6. Analysis Through the Constructional Approach Framework

Table 3 summarizes the application of the five critical elements of the constructional approach to this case, illustrating how the framework guided the functional assessment, procedure design, and progress monitoring within a time-sensitive management context.

Viewed through the five elements of the constructional approach [1,2], this case illustrates each component in practice under real-world conditions. The terminal repertoire was calm entry into the night holding area with the shift door closed, achieved with degrees of freedom preserved throughout, sufficient to restore routine husbandry management. The current relevant repertoire included the orangutan’s refusal to enter the night holding area—a functionally competent avoidance response maintained by the aversive history recently acquired in that space—alongside her established cooperative training history and waiting, eating, and interacting with caregivers just outside the doorway to Den 3, which served as the behavioral starting point for the shaping procedure. The change procedure contacted the critical consequence maintaining avoidance—distance from the night house—using that same consequence to reinforce successive approximations toward entry, while retreat remained available at every point rather than being withheld, consistent with the two safeguards against superimposition described in Section Minimizing Superimposition. Door management was treated as its own final approximation rather than a procedural afterthought. The maintaining consequences shifted across the procedure: initially the opportunity to return to the main house following each approximation, and subsequently access to the familiar social environment and the company of the male upon successful completion—a shift the team confirmed rather than assumed: when she began remaining at her station instead of retreating immediately, they continued to cue her return rather than extending criteria, verifying the change before proceeding. Progress monitoring was embedded in the team’s continuous observation of behavioral indicators across approximations—posture, movement quality, arm tension, vigilance, and orientation—with the criterion for door closure being the orangutan’s demonstration of calm, sustained presence at her usual station in Den 2.

The degrees of freedom present throughout ensured the orangutan was never required to give up access to her critical consequence. Her avoidance behavior remained effective at every point: any behavioral indicator of tension prompted a return to the main house, resetting to the last successful approximation. What changed was that calmer, more approach-oriented responses also produced the same outcome, giving her additional pathways to that same reinforcer and making avoidance functionally less necessary over the course of the procedure. The rapidity of the outcome—approximately 15 min from refusal to calm door closure—is consistent with an intervention that worked with rather than against the functional contingency organizing her behavior.

## 9. Discussion

### 9.1. Common Principles Across Case Studies

Both case studies illustrate a common set of principles that characterize effective negative reinforcement shaping for distancing-maintained behaviors in constructional programs. In each case, the critical consequence appeared to be correctly identified before the procedure began: the distance from Holger for the tiger and the distance from the night house for the orangutan. In each case, the procedure began under conditions in which the animal could readily emit desired responses, and distance was delivered promptly and contingently on those responses. In each case, the animal was never required to give up access to its critical consequence—it could always emit the historical behavior and produce the same outcome as before. In the orangutan case, time pressure and management needs were present throughout; however, the animal’s ability to return to the main house was preserved at every point in the procedure, and she exercised that option on at least one occasion, confirming that access to the critical consequence remained functional despite the operational constraints. And in each case, the outcomes were rapid and observable, with follow-up observations confirming maintenance beyond the immediate post-intervention period: no regression to aggressive responding has been observed in the tiger since March 2023, and the orangutan returned to typical cooperative shifting behavior the following day and has maintained it since.

These shared features reflect the broader principles identified across the constructional case literature [4,5,6]: utilizing NCA to identify the full contingency picture, working with functional reinforcers rather than against them, beginning under conditions that support desired responses, providing degrees of freedom that ensure genuine choice, and building behavioral repertoires with genuine assent rather than through compulsion or suppression.

### 9.2. The Speed and Efficiency of Behavior Change

Practitioners encountering negative reinforcement shaping within a constructional framework for the first time are often surprised by both the speed and efficiency of behavior change. A tiger’s aggressive responding toward a specific caregiver was resolved across 42 min of training. An orangutan shifted into a previously aversive space within 15 min. The case studies presented in this paper are consistent with outcomes reported in other applications of the constructional approach across species and contexts. A black bear accepting two people in her space—the exact condition that had occasioned aggressive displays and prevented veterinary access—within 20 min. A camel approaching a previously feared scale within a single session [4]. Miller and Abdel-Jalil describe rapid behavior change with human learners; food aversion resolved in 90 min, vacuum fear resolved in 90 min [26]. This rapidity is not accidental and is not simply a function of individual animal variability; it is consistent with what would be predicted from functionally aligned intervention within the constructional approach framework [4,5,6].

When negative reinforcement shaping is implemented within a constructional framework, the reinforcer being delivered is the one the animal’s behavior has always been organized to obtain, and learning is correspondingly efficient. Section Minimizing Superimposition describes two safeguards that keep this contingency uncontaminated by a competing one when a positive reinforcement contingency is also part of the procedure: structurally separating the two contingencies onto distinct behaviors, and preserving the animal’s ability to use previously effective behavior throughout. When these safeguards hold, the risk of superimposition—and with it the conflict, hesitation, and extinction-induced responding that superimposition can produce—is minimized. The animal is receiving exactly what its behavior has been organized to obtain, under conditions that make a less costly behavior equally effective at obtaining it. The new behavior is not replacing the old one through suppression; it is simply a more accessible pathway to the same outcome. Under these conditions, behavioral change is rapid because the intervention avoids introducing the very competition that would otherwise slow it down. This is a direct result of applying negative reinforcement within the constructional approach, guided by NCA and the principle of working with the animal’s existing functional reinforcer [1,2,4,34].

This is in direct contrast to the slow, incremental progress typical of systematic desensitization, in which each step requires extinguishing a fear response before proceeding to the next. Extinction is inherently gradual and carries with it the risks of relapse, generalization failures, and emotional side effects. Negative reinforcement shaping within a constructional framework, because it works with rather than against the functional contingencies already organizing the animal’s behavior, may sidestep these mechanisms, though the degree to which this holds will depend on implementation quality and the specific behavioral history of the individual animal [21,25].

### 9.3. Why This Is a Welfare Advancement

The welfare advantages of negative reinforcement shaping within a constructional framework over extinction-based and superimposition-based approaches can be articulated across several dimensions. Speed means less time in an aversive contingency: fewer sessions, less exposure to conditions the animal experiences as aversive, and more rapid improvement in the quality of the animal’s daily experience. Behavioral change of this kind may reflect more than response suppression: animals do not simply stop displaying fear or aggressive behavior; they begin displaying the behavioral patterns associated with nearing contingencies—approach, curiosity, and affiliation—rather than distancing ones [15,26]. The tiger began to chuff in anticipation of Holger’s arrival. The orangutan shifted calmly into a space she had refused to enter. Behavioral indicators were consistent with a shift in the functional contingency organizing each animal’s behavior: from distancing to nearing. Within a contingency-based account of emotional behavior [15], such observable changes—the tiger’s chuffing in anticipation of Holger’s arrival, the orangutan’s calm entry into a previously avoided space—reflect changes in the contingency itself, not merely changes in surface behavior.

The preservation of effective behaviors is also significant. Because the animal’s historical behavior is never extinguished but simply becomes less necessary, frustration and extinction-induced aggression may be less likely to be occasioned. This is the practical outcome of the safeguard described in Section Minimizing Superimposition and demonstrated in both case studies: previously effective behavior remains available throughout the procedure rather than being withheld, so the animal retains behavioral options and agency at every step. Genuine choice is maintained: less costly behaviors are potentiated because the procedure establishes alternative contingencies that provide the same critical consequence at less cost, rather than forcing the animal to abandon its existing behavioral options. And behavior change grounded in functional contingencies may be more durable than change achieved through suppression or superimposition, as the maintaining contingency is addressed rather than overridden—a prediction consistent with the broader constructional literature accumulated across clinical, educational, and companion animal contexts over five decades [1,2,4,7,9,10,11,12,13].

Prospective studies with systematic follow-up in zoo settings specifically are needed to extend this evidence base to managed wildlife populations. When the maintaining contingency is addressed, the new behavioral repertoire has a stable functional foundation. It bears emphasizing that these advantages are properties of negative reinforcement shaping applied within the constructional approach, not of negative reinforcement as a generic procedure. The speed, durability, and qualitative changes described here depend on the analytical and procedural commitments that the constructional framework provides.

### 9.4. Advancing Practice: A Call to the Zoo Community

The zoo animal training community has developed substantial expertise in positive reinforcement-based training over the past several decades, with clear benefits for animal welfare, staff safety, and the quality of human–animal relationships in zoological settings. A meaningful next step in zoo animal welfare practice may involve extending that expertise to include a nuanced, principled, and welfare-positive understanding of negative reinforcement within a constructional framework—not as a reluctant fallback when positive reinforcement fails, but as a primary tool when behavioral function indicates its use.

This requires a shift in how we talk about and teach behavior analysis in professional development contexts. It requires honest engagement with the limitations of traditional approaches and with the reasons those approaches can fail for specific behavioral presentations. It requires a willingness to examine our own assumptions about what “good” animal training looks like and to follow the evidence rather than the familiar.

It also requires recognizing and crediting the foundational work of the behavioral scientists and practitioners who developed these approaches, often in the face of significant professional resistance: Goldiamond, Layng, Andronis, Rosales-Ruiz, and others who built the empirical and practical foundation upon which current zoo applications rest [4]. Standing on that foundation, the zoo community is positioned to make meaningful contributions of its own to the growing body of evidence and practice.

### 9.5. Limitations and Directions for Future Research

Single-subject research designs are well-established and methodologically legitimate within behavioral science, capable of demonstrating functional relationships between procedures and outcomes with rigor [37,38]. The cases presented here, however, were not designed as research studies. Both interventions were implemented in response to real behavioral challenges requiring timely, welfare-centered solutions—the priority in each instance was the animal, not necessarily data collection. However, the procedures produced rapid, observable, and documentable outcomes, creating an opportunity to share information that may be useful to colleagues facing similar situations. Some data and video were collected on session number, duration, and behavioral indicators across the interventions, and follow-up observations confirm that behavior changes were maintained beyond the immediate post-intervention periods. These data are less rigorously controlled than those from prospectively designed single-subject studies, but they reflect the conditions under which much practitioner knowledge is generated and are consistent with the outcomes predicted by the constructional framework. The absence of prospectively collected baseline data, formal progression criteria, and inter-observer reliability assessments is an inherent limitation of the retrospective case format, and the conclusions drawn here should be interpreted accordingly.

What these points indicate is not a disqualification of the findings but a recognition that systematically collecting data when these situations arise—as they inevitably will in zoo settings—positions the field to build a genuinely rigorous evidence base from the work practitioners are already doing. The procedures described in this paper offer a foundation for that effort. The zoo community already has the settings, species diversity, and, increasingly, the procedural fluency to generate that evidence. What is needed now is the intentional collection of data as these situations present themselves, with an eye toward contributing to the empirical foundation that will make these approaches standard practice. A contemporaneous, quantified case series in tigers [20] is an encouraging sign that this work is already underway, offering multi-subject documentation of distance functioning as a reinforcer in a large felid. As this body of work expands, an important next step for the field generally will be refining shared procedural standards—including how studies report whether an effective distancing pathway remained available to the animal throughout an intervention, not only whether the terminal behavior was achieved. The present paper’s procedural sections (Section 5.3 and Section 7.2) are offered as one contribution toward that broader effort.

## 10. Conclusions

Fear and aggressive responses maintained by distancing contingencies are among the most commonly encountered and welfare-significant behavioral challenges in zoo animal care. Traditional approaches—systematic desensitization and counterconditioning—can fail to address the functional contingency maintaining these behaviors and, in such cases, may produce incomplete, temporary, or emotionally costly outcomes. Negative reinforcement shaping procedures, grounded in the constructional approach and Nonlinear Contingency Analysis, help contact critical consequences, use the animal’s own functional reinforcer to shape new behaviors, and build behavioral repertoires consistent with genuine improvement in the animal’s welfare experience.

The two case studies presented here illustrate the application of these principles in practice: a male Amur tiger whose aggressive responses toward a specific caregiver fell out of his repertoire over 42 min of training as affiliative behavior was established in its place, and an orangutan whose avoidance of a previously aversive night-holding area was resolved in approximately 15 min with calm behavioral indicators throughout. In each case, the outcome reflected the rapid behavior change observed in functionally aligned intervention, the preservation of the animal’s behavioral agency, and the qualitative change of emotional behavior that accompanies effective constructional programming.

These procedures are accessible to regular practitioners. They require skill—as all nuanced behavioral work does—but that skill is learnable and teachable. The zoo community is well-positioned to advance the application and documentation of these approaches, to build the empirical literature, and to advocate for their integration into professional training and standards. Animals in our care deserve interventions that work with, rather than against, their function and that build, rather than suppress. Negative reinforcement, properly understood and skillfully applied through the constructional approach, may be one of the most significant welfare tools we have.

## Figures and Tables

**Table 1:** Comparison of common behavioral procedures used to address fear, aggression, and avoidance-related behavior.

	Respondent Procedures		Traditional Operant Shaping		Constructional Operant Shaping	
Comparison Feature	Systematic Desensitization	Counterconditioning	Positive Reinforcement	Negative Reinforcement	Positive Reinforcement	Negative Reinforcement
Primary learning process	Graduated exposure procedure based on extinction	Respondent procedure using CS–US ^1^ pairings (proposed to substitute competing respondent relations)	Operant shaping	Operant shaping	Operant shaping	Operant shaping
Primary focus of change	Extinction of fear responses	Respondent relations elicited by a conditioned stimulus; operant repertoires are not directly addressed	Operant behavior maintained by appetitive contingencies	Operant behavior maintained by distancing contingencies	Operant behavior identified through functional assessment	Distancing-maintained operant behavior identified through functional assessment
Procedure selected because	Fear or phobic responding is observed	An aversive conditioned stimulus is identified	Practitioner judgment and/or functional assessment	Practitioner judgment and/or functional assessment	NCA identifies appetitive consequences as the critical consequence maintaining behavior	NCA identifies distancing as the critical consequence maintaining behavior
Primary consequence arranged	Graduated exposure (traditionally with relaxation)	Appetitive US paired with CS	Presentation of appetitive stimulus	Distance gained from the aversive stimulus, or its removal, reduction, postponement, or prevention	Presentation of functionally selected appetitive consequence	Distance gained from the functionally selected aversive stimulus or conditions, or their removal, reduction, postponement, or prevention
Directly contacts the contingency maintaining distancing behavior?	No	No	Variable	Variable	Yes	Yes
Role of escape/avoidance	Escape typically prevented during exposure	Escape not functionally addressed	Variable	Variable; escape may be contingent on compliance	Escape remains available and informative	Escape remains available and functions as the reinforcer
Genuine choice (degrees of freedom)	Typically absent	Typically absent	Variable	Variable	Maintained throughout by design	Maintained throughout by design
Behavioral strategy	Reduce respondent reflexes, often described as indicating fear, via extinction	Substitute competing respondent relations; the original functional relation may remain intact	Build new operant behavior	Build new operant behavior	Build new operant repertoire matched to function	Build new operant repertoire matched to function
Potential welfare considerations	May require prolonged exposure while limiting opportunities for avoidance or escape	Observable respondent behavior may decrease while the original functional relation and distancing contingency remain intact	Depends on implementation; restricted behavioral options or reinforcement available only through compliance may reduce genuine choice	Depends on implementation; restricted behavioral options or escape contingent on compliance may reduce genuine choice and increase coercion	Functional alignment, genuine choice, and repertoire expansion are structural features that support welfare	Functional alignment, preserved degrees of freedom, repertoire expansion, and planned transition toward positive reinforcement are structural features that support welfare
Representative zoo example	A syringe is gradually brought closer to an animal to prepare for an injection procedure	Food is paired with the presence of a caregiver previously associated with aversive events	Food reinforcement is used to shape progressively closer contact with a feared scale	A caregiver walks behind a group of animals to evoke forward movement	NCA identified tactile stimulation as the critical consequence; the animal can present any of several body parts to access reinforcement, ensuring genuine choice is maintained throughout the session	NCA identified distance from the caregiver as the critical consequence; multiple calm behaviors are reinforced with caregiver retreat; the animal can also retreat ensuring genuine choice is maintained across all sessions

^1^ CS: Conditioned Stimulus; US: Unconditioned Stimulus.

**Table 2:** Application of the five critical elements of the constructional approach: Case study one (Male Amur Tiger, Copenhagen Zoo).

Constructional Element	Application in Case Study One
Terminal Repertoire	Behavioral indicators consistent with relaxed, affiliative interaction with Holger sufficient to support medical training and educational presentations.
Current Relevant Repertoire	Operationally defined aggressive responses toward Holger—growling, staring, raised lip behavior, and sustained vigilance (maintained oriented body posture and directed gaze)—maintained by distancing contingencies; relaxed posture, engagement with food, and absence of vigilance responses available as shaping starting points.
Change Procedure	Negative reinforcement shaping implemented within a constructional framework, in which calm, non-aggressive responding at each successive approximation was reinforced by Holger’s contingent withdrawal, while the criterion distance at which he approached was progressively decreased across sessions. Two safeguards against superimposition were built into the procedure: food was managed separately by a second caregiver and contingent on the tiger remaining settled, keeping the two contingencies on separate behaviors; and previously effective aggressive responses remained available and were reinforced with distance if emitted, rather than withheld, preserving genuine choice throughout. The procedure contacted the maintaining contingency and progressively built the terminal repertoire.
Maintaining Consequences	Initially, distance from Holger delivered contingent on behavioral indicators consistent with relaxed responding. The absence of aggressive or vigilant responding under a criterion that had previously reliably occasioned them indicated the distancing contingency was no longer dominant, supporting the transition to food and social interaction with Holger as positive reinforcement became functionally effective.
Progress Monitoring	Video recordings and training logs documenting increasing frequency and duration of operationally defined relaxed responding across sessions, the falling out of the repertoire of aggressive responses (growling, staring, raised lip behavior, sustained vigilance), and emergence of affiliative vocalizations (chuffing) as a marker of the switchover.

**Table 3:** Application of the five critical elements of the constructional approach: Case study two (Female Bornean Orangutan, Dublin Zoo).

Constructional Element	Application in Case Study Two
Terminal Repertoire	Cued entry into the night holding area with behavioral indicators consistent with relaxed responding, allowing door closure and return to routine husbandry participation.
Current Relevant Repertoire	Avoidance of the night holding area following an aversive event—a functionally competent avoidance response maintained by the aversive history recently acquired in that space; waiting, eating, and interacting with caregivers just outside the doorway to Den 3 served as the behavioral starting point for shaping.
Change Procedure	Two distinct contingencies operated on separate behaviors, consistent with the first safeguard against superimposition described in Section Minimizing Superimposition: food was delivered contingent on arrival at successive approximation points, reinforcing movement toward the night house; the opportunity to return to the main house—distance from the night house—was delivered contingent on calm behavioral indicators while in the aversive space. Consistent with the second safeguard, retreat remained available throughout rather than being withheld; the orangutan exercised this option when a door handle movement occasioned an escape response, and this was treated as information rather than failure. The procedure contacted the maintaining contingency and progressively built the terminal repertoire
Maintaining Consequences	The critical consequence—distance from the night house—was initially maintaining avoidance and was redirected to build calm presence in the aversive space through contingent delivery of the opportunity to return to the main house. Food contingent on arrival at each approximation point maintained movement toward the space. The shift toward food functioning as an effective reinforcer was confirmed rather than assumed: when the orangutan began remaining at her station rather than retreating immediately, the team continued to cue her return rather than extending criteria, verifying behavioral change before proceeding. Upon successful completion, access to the familiar social environment and the company of the male.
Progress Monitoring	Continuous observation and video documentation of defined behavioral indicators across approximations—posture, arm tension, orientation toward caregivers, reduced vigilance (scanning toward exits, reaching through doorways), and duration of settled presence at station—with successful door closure and return to typical shifting behavior by the following day confirming outcome.

## Data Availability

Video documentation and training log data supporting the tiger case study are publicly available through the Animal Training Fundamentals online learning platform [36]. Video documentation of the orangutan session will be made available on the same platform following publication at AnimalTrainingFundamentals.com.

## Abbreviations

The following abbreviations are used in this manuscript:

NCA	Nonlinear Contingency Analysis
CAT	Constructional Aggression Treatment
CET	Constructional Exposure Therapy
CS	Conditioned Stimulus
US	Unconditioned Stimulus
